# DSCNet: Deep Skip Connections-Based Dense Network for ALL Diagnosis Using Peripheral Blood Smear Images

**DOI:** 10.3390/diagnostics13172752

**Published:** 2023-08-24

**Authors:** Manjit Kaur, Ahmad Ali AlZubi, Arpit Jain, Dilbag Singh, Vaishali Yadav, Ahmed Alkhayyat

**Affiliations:** 1 School of Computer Science and Artificial Intelligence, SR University, Warangal 506371, India; manjit@ieee.org; 2 Department of Computer Science, Community College, King Saud University, Riyadh 11421, Saudi Arabia; aalzubi@ksu.edu.sa; 3 Department of Computer Science and Engineering, Koneru Lakshmaiah Education Foundation, Vijayawada 522302, India; dr.jainarpit@gmail.com; 4 Center of Biomedical Imaging, Department of Radiology, New York University Grossman School of Medicine, New York, NY 10016, USA; 5 Research and Development Cell, Lovely Professional University, Phagwara 144411, India; 6 School of Computer and Communication Engineering, Manipal University Jaipur, Jaipur 303007, India; 7 College of Technical Engineering, The Islamic University, Najaf 7003, Iraq; ahmedalkhayyat85@gmail.com

**Keywords:** acute lymphoblastic leukemia, deep learning, skip connections, dense network, peripheral blood smear images, KL divergence loss, dropout regularization, data augmentation, diagnosis, medical imaging

## Abstract

Acute lymphoblastic leukemia (ALL) is a life-threatening hematological malignancy that requires early and accurate diagnosis for effective treatment. However, the manual diagnosis of ALL is time-consuming and can delay critical treatment decisions. To address this challenge, researchers have turned to advanced technologies such as deep learning (DL) models. These models leverage the power of artificial intelligence to analyze complex patterns and features in medical images and data, enabling faster and more accurate diagnosis of ALL. However, the existing DL-based ALL diagnosis suffers from various challenges, such as computational complexity, sensitivity to hyperparameters, and difficulties with noisy or low-quality input images. To address these issues, in this paper, we propose a novel Deep Skip Connections-Based Dense Network (DSCNet) tailored for ALL diagnosis using peripheral blood smear images. The DSCNet architecture integrates skip connections, custom image filtering, Kullback–Leibler (KL) divergence loss, and dropout regularization to enhance its performance and generalization abilities. DSCNet leverages skip connections to address the vanishing gradient problem and capture long-range dependencies, while custom image filtering enhances relevant features in the input data. KL divergence loss serves as the optimization objective, enabling accurate predictions. Dropout regularization is employed to prevent overfitting during training, promoting robust feature representations. The experiments conducted on an augmented dataset for ALL highlight the effectiveness of DSCNet. The proposed DSCNet outperforms competing methods, showcasing significant enhancements in accuracy, sensitivity, specificity, F-score, and area under the curve (AUC), achieving increases of 1.25%, 1.32%, 1.12%, 1.24%, and 1.23%, respectively. The proposed approach demonstrates the potential of DSCNet as an effective tool for early and accurate ALL diagnosis, with potential applications in clinical settings to improve patient outcomes and advance leukemia detection research.

## 1. Introduction

Acute lymphoblastic leukemia (ALL) is a devastating hematologic malignancy characterized by the abnormal proliferation of immature lymphocytes in the blood or bone marrow [1]. Early and accurate diagnosis of ALL is crucial for effective treatment and improved patient outcomes. Conventional diagnostic methods involve labor-intensive and error-prone manual examination of stained blood smear microscopic images, which can lead to delays in diagnosis and treatment initiation. As a result, researchers have turned to DL-based computer-aided diagnosis (CAD) systems to automate and enhance ALL diagnosis using peripheral blood smear (PBS) images. PBS images are routinely collected in clinical settings for the initial screening of patients suspected of having leukemia. These images contain valuable information about the morphology and distribution of blood cells, which can aid in the diagnosis of ALL.

Certainly, deep learning models have gained significant popularity and utility across a wide range of domains in recent years [2,3,4]. By leveraging the power of DL, it becomes possible to automate and enhance the accuracy of ALL diagnosis using PBS images, making it a valuable tool for hematologists and oncologists. Several studies have emerged that present innovative approaches that harness the power of DL models to aid in ALL diagnosis. Notably, Atteia proposed a hybrid DL system combining autoencoder networks for feature representation learning in the latent space with the feature extraction abilities of standard pre-trained convolutional neural networks (CNNs) [1]. Chand and Vishwakarma proposed a novel DL framework (DLF) based on convolutional neural networks for the classification of ALL, avoiding the need for feature extraction and pre-training on other databases [5]. Additionally, Masoudi presented the VKCS model, a three-stage transfer learning-based model with attention mechanisms, demonstrating promising results in diagnosing ALL [6]. Moreover, Das and Meher proposed an efficient deep CNN framework utilizing depth-wise separable convolutions and hybridizing MobileNetV2 and ResNet18 for accurate ALL detection [7].

Existing DL models for ALL diagnosis encounter various obstacles that limit their effectiveness in clinical practice. These challenges include the scarcity of comprehensive and diverse datasets, demanding the acquisition of large and representative data for training. Ensuring the generalizability of models across diverse patient populations and real-time applicability necessitates rigorous validation and performance assessment [8,9,10]. Additionally, handling data augmentation, pre-processing, and class imbalance significantly influences the model’s reliability and accuracy. To fully exploit the potential of DL in enhancing ALL diagnosis and patient outcomes, collaborative efforts are crucial to overcome these hurdles and improve the models’ practicality and clinical utility.

Despite their benefits, DL-based ALL diagnosis models have challenges, including computational complexity, sensitivity to hyperparameters, and difficulties with noisy or low-quality input images. To ensure their practical implementation in medical diagnosis, further validation in clinical settings and attempts to address these limitations are crucial. The key contributions of this paper are as follows:

1. **Deep Skip Connections-Based Dense Network (DSCNet)**: This paper proposes a novel architecture, DSCNet, specifically tailored for the diagnosis of ALL using peripheral blood smear images. DSCNet utilizes skip connections, custom image filtering, and dense blocks to capture long-range dependencies and enhance feature extraction.
2. **Custom Image Filtering**: A custom image filter is used as a pre-processing step to enhance input images and highlight relevant features. This filtering process is used to improve the quality of input data and aids the model in detecting intricate patterns associated with different stages of ALL.
3. **KL Divergence Loss Optimization**: KL divergence loss is used as an objective function for model optimization. By minimizing KL divergence between predicted and ground truth distributions, the model learns to make accurate predictions, enhancing its diagnostic accuracy.
4. **Dropout Regularization**: Dropout regularization is used to prevent overfitting during model training. This technique is used to enhance the robustness of feature representations and improve the way DSCNet generalizes unseen data.

The paper’s remaining structure is as follows: Section 2 reviews prior research. In Section 3, the proposed Deep Skip Connections-Based Dense Network (DSCNet) for ALL diagnosis is discussed. Section 4 presents analysis and experimental results. Finally, Section 5 concludes the paper and explores potential future directions.

## 2. Related Work

Genovese et al. proposed an adaptive unsharpening method combined with DL for ALL detection. The method enhanced blood sample images by improving sharpness through image processing techniques and DL. They evaluated the approach on a public database of ALL images using state-of-the-art CNNs, demonstrating the validity of their approach [11]. Rezayi et al. explored the use of artificial intelligence-oriented DL methods for timely diagnosis of ALL. They employed two famous DL networks, ResNet-50 and VGG-16, to classify leukemic cells from normal cells in microscopic images. The proposed convolutional network and various machine learning techniques achieved promising results for ALL classification, proving the potential for clinical usage in leukemia diagnosis [12]. Abunadi and Senan developed multi-method diagnostic systems for early ALL detection using DL and hybrid techniques. They proposed CNN models such as AlexNet, GoogleNet, and ResNet18, along with SVM, and achieved high accuracies in classifying ALL images. The study contributes to the development of efficient diagnostic systems for leukemia detection [13].

Ahmed and Nayak proposed the use of the VGG-19 model for the detection of lymphoblastic leukemia. By employing DL and image processing techniques, the study aimed to improve the accuracy and speed of diagnosis. The VGG-19 model with transfer learning demonstrated promising results in classifying leukemia images [14]. Ansari et al. designed a customized DL model for acute leukemia diagnosis using images of lymphocytes and monocytes. The study’s dataset, generated using GAN, contributes to the research community in developing machine learning techniques in medical research [15]. Das and Meher introduced a transfer learning-based automatic ALL detection method using the SqueezeNet model. The highly computationally efficient approach outperformed other DL models, including Xception, NasNetMobile, VGG-19, and ResNet-50, in terms of classification performance on the ALLIDB1 and ALLIDB2 databases [16].

Genovese et al. proposed a method for ALL detection using histopathological transfer learning. They trained a CNN on a histopathology database for tissue type classification and then fine-tuned it on the ALL database, achieving promising results [17]. Jawahar et al. introduced ALNett, a cluster layer deep CNN, for the classification of microscopic white blood cell images. Through its depth-wise convolution with different dilation rates and cluster layers, ALNett extracted robust local and global features, enabling accurate prediction of ALL [18]. Das et al. presented a transfer learning-based acute leukemia diagnosis model using an orthogonal softmax layer. The model, based on ResNet18, achieved superior performance compared to other models trained on small medical datasets [19].

Ghosh et al. proposed a deep CNN with an average pooling layer for simultaneous localization and classification of ALL in peripheral blood smear images. Although it had some limitations in detecting all ALL lymphocytes, it performed well in predicting whether a blood smear image belonged to an ALL patient or not [20]. Atteia et al. introduced BO-ALLCNN, a Bayesian-based optimized CNN for ALL detection in microscopic blood smear images. The CNN, optimized through Bayesian optimization, outperformed other optimized DL models in classifying ALL images [21]. Ghaderzadeh et al. developed a fast and efficient CNN model for B-ALL diagnosis and subtype classification using peripheral blood smear images. The DenseNet201-based model achieved high accuracy, sensitivity, and specificity in distinguishing ALL from benign cases and identifying ALL subtypes [22]. Gehlot et al. proposed SDCT-AuxNet${}^{\theta }$, a stain deconvolutional CNN with an auxiliary classifier for cancer diagnosis. Their novel architecture utilized stain deconvolved quantity images and a dual-classifier approach to achieve better performance [23]. Hassanien and Afify presented an ensemble strategy for detecting ALL cells versus normal WBCs using three stages: image pre-processing, deep feature extraction with a CNN–GRU–BiLSTM architecture, and classification using a softmax function and the multiclass support vector machine (MSVM) classifier [24].

Hui et al. developed an intelligent classification system for acute leukemia based on Wright–Giemsa stain blood slides. Their procedure involved image pre-processing, image segmentation using color thresholding and morphological operations, and classification of white blood cells using DL classifiers (AlexNet and GoogLeNet) [25]. Billah and Javed demonstrated the successful implementation of Bayesian convolution neural networks (BCNNs) for classifying microscopic images of blood samples (lymphocyte cells). Their BCNN-based classification procedure avoided manual feature extraction and provided useful information regarding uncertainty in predictions. The models produced high accuracy in classifying cancerous and noncancerous lymphocyte cells [26].

Jha and Dutta [27] proposed a hybrid model based on mutual information (MI) and a deep CNN classifier. This model utilized a combination of fuzzy C-means algorithms and active contour model segmentation results. After segmentation, feature extraction (statistical and the local directional pattern) was performed. Finally, the extracted features were input to a deep CNN classifier that was designed using the chronological sine cosine algorithm. Genovese et al. [28] proposed DL4ALL for the detection of ALL, which was trained using cross-dataset transfer learning. The proposed model was a multi-task learning model that transformed the given model into a multi-task classification problem. The transformed model was then trained with transfer learning, taking into account both the source and target databases simultaneously. This approach incorporated batches from the two domains, even when they were quite different.

Ullah et al. [29] provided a safe, CNN-based method for performing the diagnosis process using medical images. The proposed approach consisted of a CNN-based model, which used VGG-16 and the Efficient Channel Attention (ECA) module for better feature extraction, thereby improving feature representation and classification. The quantity and quality of training data were increased using several augmentation techniques. Mirmohammadi et al. [30] proposed a multi-phase approach for leukemia detection. The first phase involved image enhancement by converting RGB to HSV and equalizing grayscale luminance. The second phase included nuclei segmentation using fuzzy C-means clustering and noise reduction. Subsequently, features were extracted and selected, and the classification was performed using random forest. Abhishek et al. [31] proposed a novel dataset containing 500 images. This novel dataset was combined with a publicly available dataset to create a heterogeneous dataset. The dataset was used for binary and three-class classification for various CNN models.

Devi et al. [32] utilized Gaussian blurring (GB) and hue saturation value (HSV) techniques in their model, GBHSV-Leuk. They conducted a two-phase classification, blurring reflection and noise in the first phase using GB, followed by HSV-based segmentation in the second phase. Morphological methods were introduced in the second phase to enhance accuracy by separating foreground and background colors. Khandekar et al. [33] utilized artificial intelligence to automate blast cell detection through the YOLOv4 algorithm. This algorithm ensured accurate cell identification and categorization in their dataset. Additionally, they integrated a novel object detection technique, significantly boosting the precision and dependability of their proposed system. Sampathila et al. [34] introduced ALL-NET, an advanced deep learning solution for white blood cell screening using microscopic blood smear images. Unlike traditional methods relying on isolated features, ALL-NET utilized the entire dataset, resulting in more accurate identification and screening of white blood cells.

Ahmed et al. [35] introduced hybrid techniques for classifying ALL images. They extracted WBC regions using the active contour algorithm and fed these regions to CNN models. Feature selection was carried out using PCA, and deep feature maps of hybrid CNNs were employed for classification, with the assistance of classifiers like RF and XGBoost. Jiang et al. [36] combined CNN and ViT models for better image classification. Their new ViT-CNN model used both methods to understand images, resulting in improved performance. They also introduced “difference enhancement random sampling” for balanced data and noise reduction. Saeed and colleagues [37] improved EfficientNetV2S and EfficientNetB3 by adding a multi-attention layer to the final block. This made the models work better on various tasks and become less complex. They named the enhanced models Multi-Attention EfficientNetV2S and EfficientNetB3. They also used cropping and data augmentation to improve image quality and balance the dataset. Hamza et al. [38] proposed the OOLHBD-ALLD model for medical diagnosis. They used Gabor filtering to reduce noise, modified fuzzy C-means for segmentation, and EfficientNetB0 with swarm optimization for feature extraction. Class labeling was done using an attention-based long short-term memory model.

In Table 1, some recently proposed DL models are studied. These models include Deep CNN with average pooling for ALL prediction (DCNN), Bayesian-based optimized CNN (BO-ALLCNN), histopathological transfer learning (HTL), transfer learning and orthogonal softmax layer-based network (TLOS-Net), fast and efficient CNN (FCNN), adaptive unsharpening with CNN (AU-CNN), and customized DL (CDL). Each model offers specific benefits, such as high accuracy, sensitivity, and efficiency. However, they also come with their own set of challenges, including computational complexity, sensitivity to hyperparameters, and difficulties with noisy or low-quality input images. Further validation in clinical settings and attempts to address these limitations are essential for their practical implementation in medical diagnosis.

To address these issues, in this paper, we propose a DSCNet tailored for ALL diagnosis using peripheral blood smear images. The DSCNet architecture integrates skip connections, custom image filtering, KL divergence loss, and dropout regularization to enhance its performance and generalization abilities. DSCNet leverages skip connections to address the vanishing gradient problem and capture long-range dependencies, while custom image filtering enhances relevant features in the input data. KL divergence loss serves as the optimization objective, enabling accurate predictions. Dropout regularization is employed to prevent overfitting during training, promoting robust feature representations. Thus, the proposed DSCNet can efficiently handle various challenges in the existing ALL diagnosis models.

## 3. Deep Skip Connections-Based Dense Network (DSCNet) for ALL Diagnosis

Algorithm 1 outlines a comprehensive Deep Skip Connections-Based Dense Network (DSCNet) with several advanced techniques for ALL diagnosis using peripheral blood smear images. The model architecture incorporates skip connections, custom image filtering, KL divergence loss, and dropout regularization to improve its performance and generalization. The first step involves the image filtering layer, where a custom image filtering operation is applied to enhance the input images. This pre-processing step helps to highlight relevant features and improve the quality of the input data. The model then proceeds through multiple layers, including convolutional blocks and dense blocks. The convolutional blocks perform convolution operations on the filtered images, followed by activation with a non-linear function (e.g., ReLU). The dense blocks utilize dense connections, combining the feature maps from previous layers with the filtered input images through concatenation.

Skip connections are employed to enable information flow across different layers of the network. This technique helps mitigate the vanishing gradient problem and allows the model to capture long-range dependencies, leading to improved performance. To optimize the model, the KL divergence loss is defined as the objective function. This loss function measures the difference between the predicted probability distribution and the ground truth distribution of ALL subtypes. By minimizing the KL divergence, the model learns to make accurate predictions. To prevent overfitting during training, dropout regularization is introduced. This technique randomly deactivates a fraction of neurons during each training iteration, which promotes more robust feature representations and enhances generalization to unseen data. The model culminates with a final softmax [40,41,42] classifier, producing a probability distribution over different ALL subtypes for each input image. This enables the model to classify the images into specific diagnostic categories with associated probabilities.

The integration of these techniques makes the deep dense model more robust, accurate, and efficient in diagnosing ALL based on peripheral blood smear images. By leveraging skip connections, custom image filtering, KL divergence loss, and dropout regularization, the model can better handle challenges such as noisy data, complex feature interactions, and overfitting. The goal is to provide an effective tool for early and accurate ALL diagnosis, contributing to improved patient outcomes and facilitating medical research in the field of leukemia detection.
**Algorithm 1** Deep Skip Connections-Based Dense Network (DSCNet) for ALL diagnosis1:**Input**: $X\in R^{n\times m\times c}$, where n,m are image dimensions and *c* is the number of channels2:**Image Filtering Layer**: $X_{\mathrm{filtered}}=\mathrm{filter}\left(X\right)$, where filter is a custom image filtering operation to enhance the images3:**Layer 1 (Convolutional Block)**: $Z^{\left(1\right)}=\sigma \left(W^{\left(1\right)}*X_{\mathrm{filtered}}+b^{\left(1\right)}\right)$, where ∗ denotes the convolution operation and σ is the activation function4:**Layer 2 (Dense Block)**: $Z^{\left(2\right)}=\sigma \left(\left[Z^{\left(1\right)},X_{\mathrm{filtered}}\right]\right)$, where $\left[Z^{\left(1\right)},X_{\mathrm{filtered}}\right]$ denotes concatenation of feature maps from Layer 1 and filtered input image5:**Layer 3 (Convolutional Block)**: $Z^{\left(3\right)}=\sigma \left(W^{\left(3\right)}*Z^{\left(2\right)}+b^{\left(3\right)}\right)$6:**Layer 4 (Dense Block)**: $Z^{\left(4\right)}=\sigma \left(\left[Z^{\left(3\right)},Z^{\left(2\right)}\right]\right)$7:**Layer 5 (Convolutional Block)**: $Z^{\left(5\right)}=\sigma \left(W^{\left(5\right)}*Z^{\left(4\right)}+b^{\left(5\right)}\right)$8:**Layer 6 (Dense Block)**: $Z^{\left(6\right)}=\sigma \left(\left[Z^{\left(5\right)},Z^{\left(4\right)}\right]\right)$9:**Layer 7 (Convolutional Block)**: $Z^{\left(7\right)}=\sigma \left(W^{\left(7\right)}*Z^{\left(6\right)}+b^{\left(7\right)}\right)$10:**Layer 8 (Dense Block)**: $Z^{\left(8\right)}=\sigma \left(\left[Z^{\left(7\right)},Z^{\left(6\right)}\right]\right)$11:**Layer 9 (Convolutional Block)**: $Z^{\left(9\right)}=\sigma \left(W^{\left(9\right)}*Z^{\left(8\right)}+b^{\left(9\right)}\right)$12:**Layer 10 (Dense Block)**: $Z^{\left(10\right)}=\sigma \left(\left[Z^{\left(9\right)},Z^{\left(8\right)}\right]\right)$13:**Layer 11 (Convolutional Block)**: $Z^{\left(L\right)}=\sigma \left(W^{\left(L\right)}*Z^{\left(L-1\right)}+b^{\left(L\right)}\right)$14:**Layer 12 Flatten Layer**: $Z_{\mathrm{flatten}}=\mathrm{flatten}\left(Z^{\left(L\right)}\right)$, which reshapes the feature maps into a vector15:**Layer 13 Fully Connected with Dropout**: $Z^{\left(13\right)}=\sigma \left(W^{\left(L+1\right)}Z_{\mathrm{flatten}}+b^{\left(L+1\right)}\right)$, with dropout regularization applied during training16:**Layer 14 Fully Connected with Dropout**: $Z^{\left(14\right)}=\sigma \left(W^{\left(L+2\right)}Z^{\left(13\right)}+b^{\left(L+2\right)}\right)$, with dropout regularization applied during training17:**Final Layer (Softmax Classifier)**: $\hat{Y}=\mathrm{softmax}\left(Z^{\left(14\right)}\right)$

### 3.1. Training Process of DSCNet

In the training process, the model uses the Kullback–Leibler (KL) divergence loss $L_{\mathrm{KL}}\left(Y,\hat{Y}\right)$ as the objective function to optimize the parameters (weights and biases) of the model. The KL divergence loss measures the divergence between the predicted probability distribution $\hat{Y}$ and the ground truth distribution *Y*.

The overall training objective becomes:

$$L_{\mathrm{total}}\left(Y,\hat{Y}\right)=L_{\mathrm{KL}}\left(Y,\hat{Y}\right)+\lambda \cdot L_{\mathrm{reg}}\left(W\right),$$

where $L_{\mathrm{reg}}\left(W\right)$ represents a regularization term on the model weights *W* to prevent overfitting, and λ is the regularization parameter.

By minimizing the KL divergence loss and the regularization term, the model can learn to classify ALL images accurately while avoiding overfitting. The trained model can then be used to predict the class label of new peripheral blood smear images and aid in the diagnosis of ALL.

Algorithm 2 outlines the training process for a DSCNet designed for ALL diagnosis. The model takes as input a set of training data consisting of image–label pairs, where each image represents a peripheral blood smear image for ALL diagnosis. The algorithm starts by randomly initializing the model parameters, including weights and biases, and defining the KL divergence loss as the optimization objective. The training process involves multiple epochs, and within each epoch, the data are divided into mini-batches to reduce memory usage and accelerate convergence. The forward pass is performed through the model architecture, which includes image filtering to enhance input images, followed by convolutional and dense blocks with skip connections. This process generates predicted probabilities for the mini-batch. The KL divergence loss is then computed between the predicted and true label distributions, and the gradients are calculated during the backward pass through back-propagation. An optimizer is utilized to update the model parameters, aiming to minimize the loss and improve the model’s ability to accurately diagnose ALL. This training loop is repeated for the specified number of epochs, ultimately fine-tuning the deep dense model to effectively detect ALL in peripheral blood smear images.
**Algorithm 2** Training the Deep Skip Connections-Based Dense Network (DSCNet)1:**Input**: Training data ${\left\{\left(X_{i},Y_{i}\right)\right\}}_{i=1}^{N}$, where $X_{i}\in R^{n\times m\times c}$ is the input image, $Y_{i}$ is the corresponding label, and *N* is the number of training samples2:**Initialize**: Randomly initialize model parameters $W^{\left(l\right)}$ and $b^{\left(l\right)}$ for each layer *l*, including filters for the image filtering layer3:**Define Loss Function**: KL divergence loss $L=\frac{1}{N}\sum_{i=1}^{N}\mathrm{KL}\left(Y_{i},{\hat{Y}}_{i}\right)$, where KL is the Kullback–Leibler divergence between ground truth label distribution $Y_{i}$ and predicted label distribution ${\hat{Y}}_{i}$4:**Define Optimizer**: Initialize optimizer parameters (e.g., learning rate, momentum, etc.)5:**Training Loop**:6:**for** epoch←1 **to** $N_{\mathrm{epochs}}$ **do**7:   **for** batch←1 **to** $N_{\mathrm{batches}}$ **do**8:     **Mini-batch Data**: Sample a mini-batch of training data $\left\{\left(X_{\mathrm{batch}},Y_{\mathrm{batch}}\right)\right\}$9:     **Forward Pass**:10:      Perform image filtering to obtain $X_{\mathrm{filtered}}$11:      Perform forward pass through all layers until the final softmax classifier to obtain predicted probabilities ${\hat{Y}}_{\mathrm{batch}}$12:     **Compute Loss**:13:      Compute KL divergence loss using ${\hat{Y}}_{\mathrm{batch}}$ and $Y_{\mathrm{batch}}$14:     **Backward Pass**:15:      Compute gradients of the loss with respect to all model parameters using back-propagation16:     **Update Model Parameters**:17:      Use the optimizer to update the model parameters $W^{\left(l\right)}$ and $b^{\left(l\right)}$ for each layer *l*18:   **end for**19:**end for**

### 3.2. Hyperparameters of DSCNet

To specify the hyperparameters of DSCNet for ALL diagnosis, we need to define the values for various parameters that influence the model’s training and performance. The choice of hyperparameters depends on the dataset, model complexity, and computational resources. In this paper, we have defined the hyperparameter values using a trial-and-error method as follows:

1. **Learning Rate**: The learning rate controls the step size during model parameter updates. An appropriate learning rate is crucial for successful training without overshooting or becoming stuck in local minima. Learning Rate = 0.001.
2. **Number of Epochs**: The number of epochs determines how many times the entire dataset is passed through the model during training. Number of Epochs = 50.
3. **Batch Size**: The batch size specifies the number of training examples in each mini-batch. Larger batch sizes may increase training speed, but too large a batch can lead to memory issues. Batch Size = 32.
4. **Regularization Parameter (λ)**: The regularization parameter controls the strength of regularization, preventing overfitting by penalizing large weights. λ = 0.01.
5. **Dropout Rate**: The dropout rate determines the fraction of neurons dropped during training, promoting robustness. We have used two dropout rates—0.3 and 0.2, respectively.

## 4. Performance Analysis

The experiments were performed on MATLAB 2022a, utilizing the DL toolbox, on a high-performance ThinkStation P360 Tower Workstation. The workstation is equipped with an Intel^®^ Core™ i9-12900 vPro^®^ Processor, an NVIDIA^®^ RTX™ A2000 12 GB GPU, and 64 GB of DDR5 4400 MHz RAM. This powerful hardware setup enabled faster training and inference times, making it suitable for running complex DL models like the DSCNet for ALL diagnosis on peripheral blood smear images. To evaluate the performance of DSCNet, other loss functions, i.e., multi-class cross-entropy loss (MCELoss) and sparse multi-class cross-entropy loss (SMLoss) were also used.

### 4.1. Dataset

The dataset for ALL [43] consists of 20,000 images, divided into four classes. Each class has 5000 images, making the dataset balanced with an equal number of samples for each class. The class labels and their corresponding descriptions are as follows:

1. **ALL_benign**: Benign—Represents images of peripheral blood smears that are classified as benign, meaning there are no signs of leukemia.
2. **ALL_early**: Early—Contains images representing the early stages of ALL.
3. **ALL_pre**: Pre—Includes images of peripheral blood smears from patients in the pre-ALL stage, indicating a progression towards leukemia.
4. **ALL_pro**: Pro—Comprises images from patients in the pro-ALL stage, representing a more advanced state of ALL.

**Figure 1:**
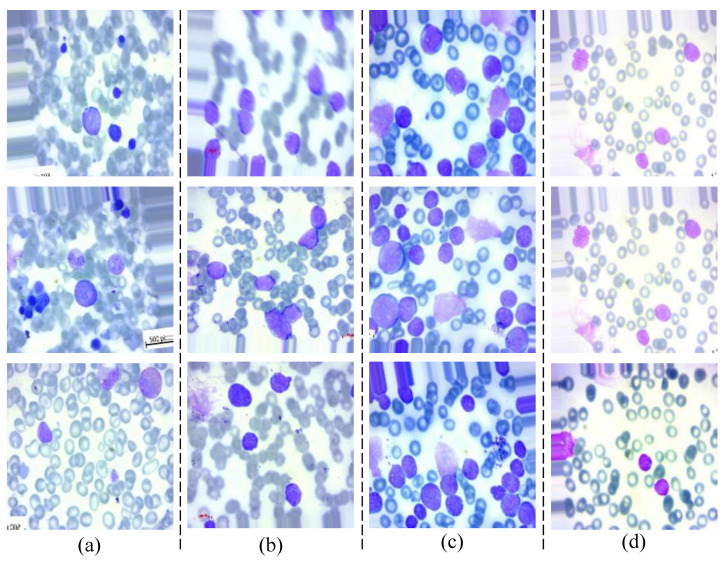
Sample images in the ALL dataset: (**a**) ALL_benign, (**b**) ALL_early, (**c**) ALL_pre, and (**d**) ALL_pro.

Figure 1 showcases sample images from the ALL dataset, representing different stages of the disease: benign, early, pre, and pro. Despite having distinct complex features, the images appear inherently similar, making the diagnostic process challenging. Accurate differentiation between normal cells and various stages of cancer is crucial for effective treatment.

### 4.2. Data Augmentation

Data augmentation is a powerful technique used to increase the diversity and size of a training dataset by applying various transformations to the original images. This helps the model generalize better and improves its performance. In this paper, the following data augmentation approaches are used.

1. **Horizontal and Vertical Flips**: This data augmentation technique involves flipping the image horizontally or vertically. By doing so, the model is exposed to different orientations of objects in the image, which enhances its robustness to variations in object direction.
2. **Random Rotations**: Random rotations are applied to the image by rotating it by a random angle. This approach allows the model to learn from images with various angles, making it more capable of handling rotated images during inference. By augmenting the dataset with rotated versions of the original images, the model gains the ability to recognize objects and patterns from different perspectives.
3. **Random Crop and Resize**: With random crop and resize, a portion of the image is randomly cropped and then resized back to the original size. This technique enables the model to focus on different regions of the image during training, promoting robustness and reducing sensitivity to the precise object location. By training on diverse crops, DSCNet learns to recognize important features that may appear in different parts of the image, improving its generalization performance on unseen data.
4. **Color Jittering**: Color jittering involves randomly modifying the color channels of the image, including altering the hue, saturation, and brightness. This augmentation introduces variations in color, making the model more resilient to changes in lighting conditions and color distributions in the dataset. By simulating different lighting conditions and color shifts, the model becomes more adaptable to real-world scenarios where images may have varying color casts or brightness levels.

These data augmentation techniques effectively augment the training dataset, enabling the DSCNet to learn more generalized and discriminative features, ultimately leading to improved accuracy in ALL diagnosis from peripheral blood smear images. By combining these data augmentation techniques, a larger and more diverse dataset is obtained. This augmented dataset can be used for training DSCNet, enhancing its ability to recognize different patterns and generalize well to unseen data.

### 4.3. Training and Validation Loss Analysis

**Figure 2:**
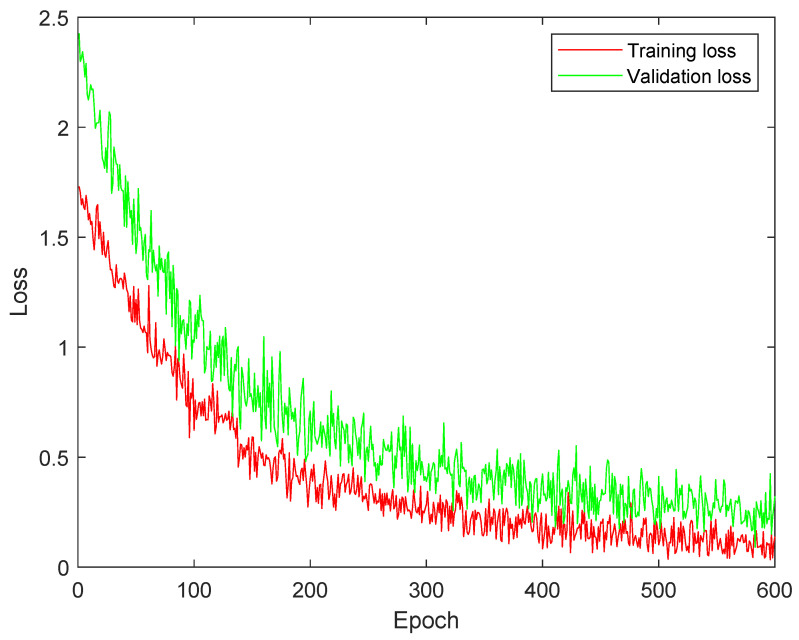
Training and validation analysis of DSCNet with multi-class cross-entropy loss (MCELoss).

Figure 2 illustrates the training and validation analysis of DSCNet using MCELoss. It demonstrates the difference between training and validation loss. The presence of a significant gap between the two curves indicates overfitting, meaning the model is performing well on the training data but struggling to generalize to new, unseen data represented by the validation set. Additionally, the slow convergence of the curves suggests that the model’s learning process is taking a considerable amount of time.

**Figure 3:**
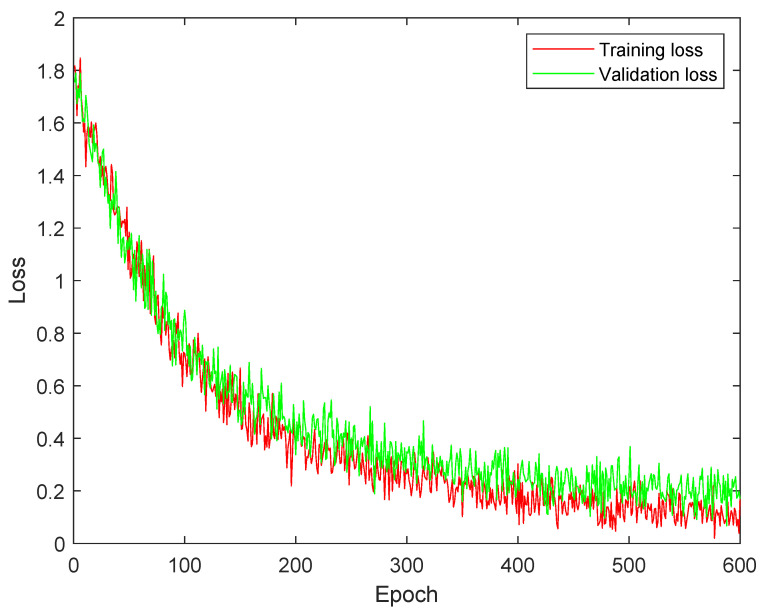
Training and validation analysis of DSCNet with sparse multi-class cross-entropy loss (SMLoss).

Figure 3 shows the training and validation analysis of DSCNet utilizing SMLoss. Compared to Figure 2, it shows a reduced overfitting impact. This improvement suggests that the model’s performance on the validation set is closer to its performance on the training set, indicating better generalization. However, despite the progress made, there is still room for further improvement to achieve better training results. This implies that the model’s performance can be enhanced by using KLLoss.

**Figure 4:**
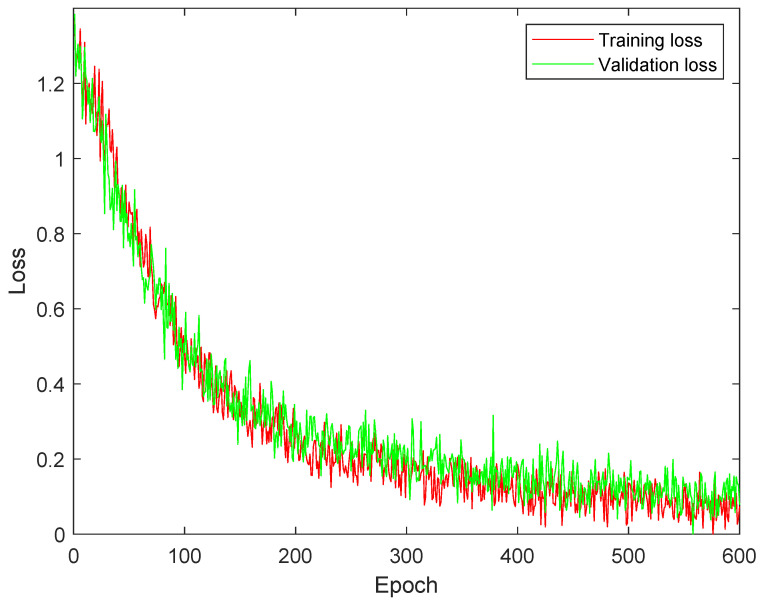
Training and validation analysis of DSCNet with Kullback–Leibler divergence loss (KLLoss).

Figure 4 demonstrates the training and validation analysis of DSCNet using KLLoss. Comparing this to Figure 2 and Figure 3, it becomes evident that the model achieves remarkable performance improvements. Convergence of the training and validation curves appears to be much better, indicating that the model is learning more efficiently. Moreover, there is a significant reduction in the impact of overfitting, suggesting that the model is generalizing well to unseen data. The use of KLLoss seems to have resulted in substantial enhancements in the model’s overall performance and training stability. This indicates that KLLoss is a promising choice for optimizing DSCNet and achieving better results.

### 4.4. Comparative Analysis

#### 4.4.1. Without Augmented Dataset

**Figure 5:**
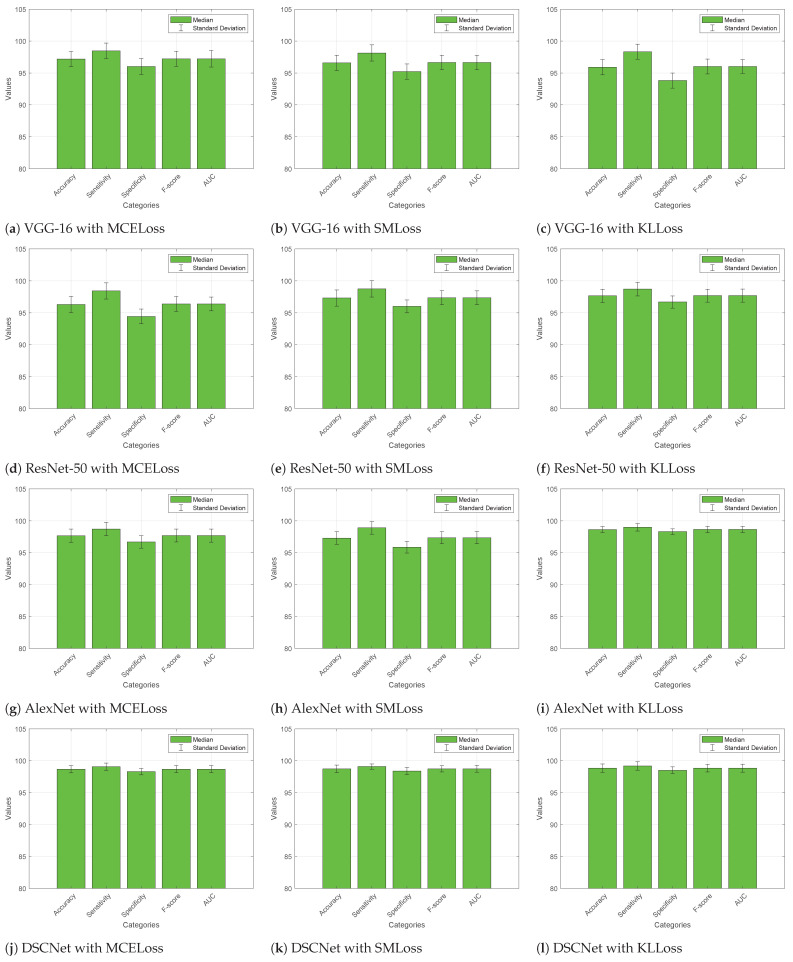
Comparative analysis of DSCNet with other models with various loss functions on the augmented ALL dataset.

Figure 5 presents the quantitative performance metrics for different models evaluated using various loss functions on the ALL dataset without data augmentation. The metrics measured are accuracy, sensitivity (also known as true positive rate or recall), specificity (true negative rate), F-score (harmonic mean of precision and recall), and area under the curve (AUC) for each model. It is found that VGG-16 with MCELoss achieved an average accuracy of 97.18%, sensitivity of 98.48%, specificity of 96.01%, F-score of 97.23%, and an AUC of 97.23%. VGG-16 with SMLoss showed an average accuracy of 96.59%, sensitivity of 98.14%, specificity of 95.22%, F-score of 96.66%, and an AUC of 96.65%. VGG-16 with KLLoss yielded an average accuracy of 95.91%, sensitivity of 98.34%, specificity of 93.82%, F-score of 96.02%, and an AUC of 96.02%. ResNet-50 with MCELoss achieved an average accuracy of 96.29%, sensitivity of 98.42%, specificity of 94.43%, F-score of 96.38%, and an AUC of 96.38%. ResNet-50 with SMLoss demonstrated an average accuracy of 97.31%, sensitivity of 98.74%, specificity of 96.01%, F-score of 97.36%, and an AUC of 97.36%. ResNet-50 with KLLoss achieved an average accuracy of 97.66%, sensitivity of 98.71%, specificity of 96.69%, F-score of 97.69%, and an AUC of 97.69%.

AlexNet with MCELoss yielded an average accuracy of 97.30%, sensitivity of 98.90%, specificity of 95.85%, F-score of 97.35%, and an AUC of 97.35%. AlexNet with SMLoss showed an average accuracy of 98.70%, sensitivity of 98.98%, specificity of 98.43%, F-score of 98.71%, and an AUC of 98.70%. AlexNet with KLLoss achieved an average accuracy of 98.63%, sensitivity of 98.99%, specificity of 98.30%, F-score of 98.64%, and an AUC of 98.64%. DSCNet with MCELoss demonstrated an average accuracy of 98.67%, sensitivity of 99.07%, specificity of 98.29%, F-score of 98.68%, and an AUC of 98.68%. DSCNet with SMLoss yielded an average accuracy of 98.73%, sensitivity of 99.09%, specificity of 98.39%, F-score of 98.74%, and an AUC of 98.74%. DSCNet with KLLoss achieved an average accuracy of 98.84%, sensitivity of 99.18%, specificity of 98.51%, F-score of 98.85%, and an AUC of 98.84%.

Overall, the models trained with KLLoss show promising performance, with high accuracy, sensitivity, specificity, F-score, and AUC. DSCNet with KLLoss achieves remarkable performance compared to the other competitive models. It demonstrates the highest median accuracy, sensitivity, specificity, F-score, and AUC among all evaluated models.

#### 4.4.2. With Augmented Dataset

**Figure 6:**
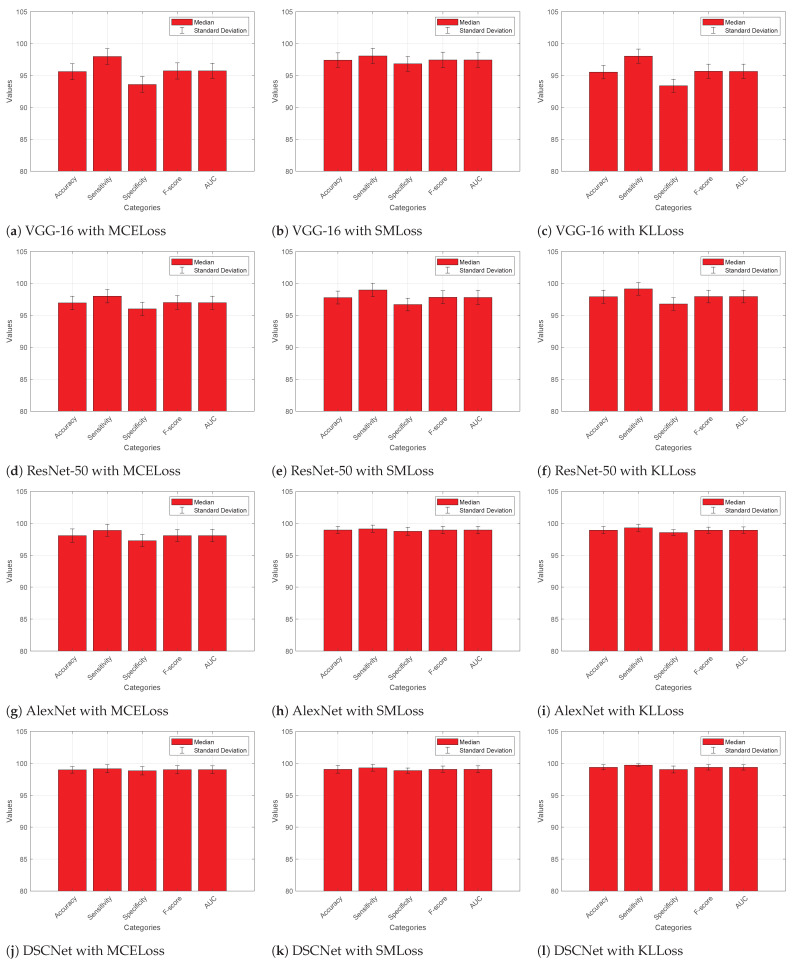
Performance analysis of competitive models with different loss functions on the ALL dataset without data augmentation.

Figure 6 presents the quantitative performance metrics for different models evaluated using various loss functions on the ALL dataset with data augmentation. It is found that VGG-16 with MCELoss achieved an average accuracy of 95.59%, sensitivity of 97.96%, specificity of 93.57%, F-score of 95.71%, and an AUC of 95.71%. VGG-16 with SMLoss showed an average accuracy of 97.39%, sensitivity of 98.04%, specificity of 96.81%, F-score of 97.42%, and an AUC of 97.42%. VGG-16 with KLLoss yielded an average accuracy of 95.51%, sensitivity of 98.03%, specificity of 93.38%, F-score of 95.65%, and an AUC of 95.64%. ResNet-50 with MCELoss achieved an average accuracy of 96.93%, sensitivity of 97.98%, specificity of 95.99%, F-score of 96.98%, and an AUC of 96.97%. ResNet-50 with SMLoss demonstrated an average accuracy of 97.77%, sensitivity of 98.96%, specificity of 96.68%, F-score of 97.81%, and an AUC of 97.80%. ResNet-50 with KLLoss achieved an average accuracy of 97.89%, sensitivity of 99.13%, specificity of 96.76%, F-score of 97.93%, and an AUC of 97.93%.

AlexNet with MCELoss yielded an average accuracy of 98.06%, sensitivity of 98.90%, specificity of 97.28%, F-score of 98.08%, and an AUC of 98.08%. AlexNet with SMLoss showed an average accuracy of 98.94%, sensitivity of 99.13%, specificity of 98.76%, F-score of 98.95%, and an AUC of 98.94%. AlexNet with KLLoss achieved an average accuracy of 98.91%, sensitivity of 99.30%, specificity of 98.55%, F-score of 98.93%, and an AUC of 98.92%. DSCNet with MCELoss demonstrated an average accuracy of 98.98%, sensitivity of 99.14%, specificity of 98.84%, F-score of 98.99%, and an AUC of 98.99%. DSCNet with SMLoss yielded an average accuracy of 99.06%, sensitivity of 99.29%, specificity of 98.85%, F-score of 99.07%, and an AUC of 99.07%. DSCNet with KLLoss achieved an average accuracy of 99.37%, sensitivity of 99.71%, specificity of 99.03%, F-score of 99.37%, and an AUC of 99.37%. Overall, DSCNet with KLLoss appears to exhibit the highest performance, with excellent convergence, minimal overfitting, and superior accuracy in comparison to the other models and loss functions.

### 4.5. Discussion

In this study, we conducted a comparative analysis of various deep learning (DL) models for the diagnosis of ALL using peripheral blood smear images. The models considered for comparison included DCNN, BO-ALLCNN, SqueezeNet, HTL, TLOS-Net, VGG-19, ResNet-50, VGG-16, FCNN, CNN, AU-CNN, BCNN, AlexNet, GoogLeNet, CDL, CNN–GRU–BiLSTM, ALNett, and SDCT-AuxNet${}^{\theta }$. Each model had its strengths and limitations in terms of accuracy, computational efficiency, sensitivity to hyperparameters, and ability to handle noisy or low-quality input images.

Among the models, DSCNet stood out, with its novel architecture specifically designed for ALL diagnosis. By incorporating skip connections, custom image filtering, and dense blocks, DSCNet achieved improved performance in detecting ALL stages from peripheral blood smear images. The custom image filtering operation enhanced the quality of input data, while the KL divergence loss optimization enabled accurate predictions, contributing to better diagnostic accuracy.

Table 2 presents a comprehensive comparison of different leukemia detection methods along with their performance metrics. The methods are evaluated in terms of accuracy, sensitivity, specificity, and F-score. Jha and Dutta’s method [27] achieved an accuracy of 98.7% using a chronological SCA-based deep CNN on the ALL-IDB2 dataset. Genovese et al.’s DL4ALL [28] achieved an accuracy of 97.85% with high sensitivity and specificity, using a multi-task learning model trained on ADP and C_NMC_2019 datasets. Ullah et al.’s VGG-16 [29] attained an accuracy of 91.1% with good sensitivity and specificity on the C_NMC_2019 dataset. Mirmohammadi et al.’s RF classifier [30] achieved an accuracy of 98.22% on the Isfahan University of Medical Sciences dataset. Abhishek et al.’s CNN model [31] reached an accuracy of 97% on a novel dataset and ALL-IDB. Devi et al.’s GBHSV-Leuk [32] obtained an accuracy of 95.41% with balanced sensitivity and specificity on a private dataset and ALL-IDB1. Khandekar et al.’s YOLOv4 [33] achieved an accuracy of 92% with high sensitivity and specificity on the ALL_IDB1 and C_NMC_2019 datasets. Sampathila et al.’s ALL-NET [34] reached an accuracy of 95.54% with balanced sensitivity and specificity on the ALL challenge dataset and C_NMC_2019. Hamza et al.’s ODLHBD-ALLD [38] attained an accuracy of 96.97% with balanced sensitivity and specificity on the ALL_IDB1 dataset. Additionally, the proposed DSCNet with KLLoss achieved exceptional performance, with an accuracy of 99.37%, very high sensitivity and specificity, and a remarkable F-score of 99.37%, showcasing its effectiveness on the ALL dataset.

The comparison with other models reveals that DSCNet offers a competitive edge in ALL diagnosis, outperforming several existing models. While various DL models show promising results in ALL classification, DSCNet’s unique architecture and advanced techniques allows it to handle challenges like noisy data and overfitting more effectively. Overall, the proposed DSCNet demonstrated its potential as a robust and accurate tool for ALL diagnosis from peripheral blood smear images. The model’s enhanced feature extraction, optimization, regularization, and data augmentation techniques contributed to its superior performance, providing valuable insights for medical research in the field of leukemia detection and potentially improving patient outcomes through early and accurate diagnosis.

## 5. Conclusions

This paper presented a Deep Skip Connections-Based Dense Network (DSCNet) for the diagnosis of ALL using peripheral blood smear images. The DSCNet architecture incorporated skip connections, custom image filtering, KL divergence loss, and dropout regularization to enhance its performance and generalization abilities. Through the integration of skip connections, the model effectively mitigated the vanishing gradient problem and captured long-range dependencies, resulting in improved performance compared to traditional architectures. The custom image filtering operation in the pre-processing step highlighted relevant features, enhancing the quality of input data and facilitating the model’s ability to detect intricate patterns.

Utilizing KL divergence loss for optimization, the proposed DSCNet accurately predicted probability distributions of ALL subtypes, thereby enhancing diagnostic accuracy. Overfitting was effectively countered through dropout regularization, yielding a robust model. Generalization of DSCNet was further improved via data augmentation. Experimental findings underscored DSCNet’s superiority over competing models, resulting in substantial improvements in accuracy, sensitivity, specificity, F-score, and AUC by 1.25%, 1.32%, 1.12%, 1.24%, and 1.23%, respectively.

The proposed DSCNet serves as a powerful tool for early and accurate diagnosis of ALL based on peripheral blood smear images, supporting medical professionals in making informed decisions. Its robustness, accuracy, and efficiency make it a valuable asset in the field of leukemia detection, contributing to improved patient outcomes and facilitating medical research. Future work may explore the potential of DSCNet in other medical image classification tasks and investigate ways to adapt and optimize the model for different types of leukemia and blood-related disorders.

The efficacy of DSCNet is intricately linked to caliber and diversity of the training dataset, while the intricate architecture could pose challenges in terms of interpretability. Furthermore, resource-intensive demands for training and inference might hinder widespread accessibility, and its current scope is confined to ALL diagnosis. In the future, there are important directions we can explore to make DSCNet even better. We could work on making it easier to understand how the model makes decisions, combining different kinds of medical information to improve its accuracy, using it for more types of diagnoses, making it work faster for quick results, reducing any unfair influences in its results, and testing it in real medical settings to prove how well it works. By doing this, we can make DSCNet stronger and contribute to making medical tests better for people’s health.

## Figures and Tables

**Table 1:** Comparative analysis of the DL-based ALL diagnosis models.

Ref.	Year	Model Name	Benefits	Unsolved Challenges
[20]	2017	DCNN	Simultaneous localization and classification of ALL in peripheral blood smear images	Limitations in detecting all ALL lymphocytes
[21]	2020	BO-ALLCNN	Improved performance in ALL image classification	Limitations or trade-offs in employing BO not mentioned
[23]	2020	SDCT-AuxNet${}^{\theta }$	Improved performance with stain deconvolved quantity images and dual-classifier approach	Generalizability to other cancer types or datasets not addressed
[16]	2021	SqueezeNet	Highly computationally efficient approach with superior performance	Limited depth and sensitivity to noisy data
[17]	2021	HTL	Promising results in ALL detection after fine-tuning on ALL database	May perform poorly for images with poor visibility
[19]	2021	TLOS-Net	Efficient ALL detection on small medical datasets using ResNet18	Potential computational overhead, requirement of optimal tuning, and sensitivity to certain hyperparameters
[14]	2021	VGG-19	Improved accuracy and speed of leukemia image classification	High computational complexity and memory requirements
[12]	2021	ResNet-50 & VGG-16	Promising results for ALL classification and potential clinical usage in leukemia diagnosis	Need for further validation in clinical settings not addressed, high computational complexity and memory requirements
[22]	2022	FCNN	High accuracy, sensitivity, and specificity in distinguishing ALL from benign cases	Potential computational overhead, requirement of optimal tuning, and sensitivity to certain hyperparameters
[39]	2022	CNN	High accuracy and specificity in distinguishing ALL from benign cases	May lead to overfitting, requirement of optimal tuning, and sensitivity to certain hyperparameters
[11]	2021	AU-CNN	Enhancement of blood sample images, improved sharpness, and potential for accurate ALL detection	Need for further validation in clinical settings not addressed, high computational complexity and memory requirements
[26]	2022	BCNN	High accuracy in classifying cancerous and noncancerous lymphocyte cells	Requirement of optimal tuning and sensitivity to certain hyperparameters
[13]	2022	AlexNet, GoogLeNet, & ResNet18	High accuracies in ALL image classification, contributing to efficient diagnostic systems	High computational complexity, memory requirements, and challenges with noisy or low-quality input images
[15]	2023	CDL	Contribution to medical research with GAN-generated dataset	Biases in the generated data and potential lack of diversity in samples
[24]	2023	CNN–GRU–BiLSTM	High accuracy and sensitivity in ALL cell detection	Sensitivity to specific hyperparameters and the need for careful tuning
[18]	2023	ALNett	Robust local and global feature extraction for accurate ALL prediction	Challenges with noisy or low-quality input images
[25]	2023	AlexNet & GoogLeNet	High accuracy in classifying white blood cells for ALL detection	GoogLeNet’s complex architecture leads to high memory consumption and makes fine-tuning challenging
[29]	2021	VGG-16	Provides better robustness and adaptability	High computational complexity and memory requirements
[36]	2021	ViT-CNN	Improved accuracy by using two different feature extraction methods simultaneously	High computational complexity and memory requirements
[34]	2022	ALLNET	Can be used during peripheral or complete blood count test	High computational complexity and memory requirements
[31]	2022	CNN model	Heterogeneous dataset utilized for binary and three-class classification	Potential lack of diversity in data
[38]	2022	ODLHBD-ALLD	Better accuracy by incorporating several state-of-the-art techniques	Potential computational overhead, trained & tested on a small dataset
[35]	2023	Hybrid CNN	Improved accuracy and fusion of different CNN models	Potential computational overhead, requirement of optimal tuning
[32]	2023	GBHSV-Leuk	Improved prediction accuracy	May lead to overfitting and sensitivity to certain hyperparameters
[28]	2023	DL4ALL	Detects ALL even if manual labels are not used for the source domain	Computationally expensive

**Table 2:** Performance comparison of ALL diagnosis models.

Ref.	Method	Accuracy	Sensitivity	Specificity	F-Score	Dataset
[27]	SCA-based deep CNN	98.7	-	-	-	ALL-IDB2
[28]	DL4ALL	97.85	95.81	98.79	-	ADP and C_NMC_2019
[29]	VGG-16	91.1	92.31	90.25	90.65	C_NMC_2019
[30]	RF Classifier	98.22	-	-	-	Isfahan Univ. of Med. Sci.
[31]	VGG-16 DenseNet & SVM	97	-	-	-	Novel dataset and ALL-IDB
[32]	GBHSV-Leuk	95.41	87.75	95.81	91.61	Private dataset and ALL-IDB1
[33]	YOLOv4	92	96	91	92	ALL_IDB1 and C_NMC_2019
[34]	ALL-NET	95.54	95.91	95.81	95.43	ALL and C_NMC_2019
[38]	ODLHBD-ALLD	96.97	96.88	96.88	96.96	ALL_IDB1
**Proposed**	**DSCNet**	**99.37 ± 0.40**	**99.71 ± 0.21**	**99.03 ± 0.55**	**99.37 ± 0.43**	ALL dataset

## Data Availability

The used dataset is freely available at https://www.kaggle.com/datasets/obulisainaren/multi-cancer, accessed on 10 August 2023.
